# Epstein-Barr Virus Induces Erosive Arthritis in Humanized Mice

**DOI:** 10.1371/journal.pone.0026630

**Published:** 2011-10-19

**Authors:** Yoshikazu Kuwana, Masami Takei, Misako Yajima, Ken-Ichi Imadome, Hirotake Inomata, Masaaki Shiozaki, Natsumi Ikumi, Takamasa Nozaki, Hidetaka Shiraiwa, Noboru Kitamura, Jin Takeuchi, Shigemasa Sawada, Naoki Yamamoto, Norio Shimizu, Mamoru Ito, Shigeyoshi Fujiwara

**Affiliations:** 1 Division of Hematology and Rheumatology, Department of Medicine, Nihon University School of Medicine, Tokyo, Japan; 2 Department of Microbiology, Yong Loo Lin School of Medicine, National University of Singapore, Singapore; 3 Department of Infectious Diseases, National Research Institute for Child Health and Development, Tokyo, Japan; 4 Department of Virology, Division of Medical Science, Medical Research Institute, Tokyo Medical and Dental University, Tokyo, Japan; 5 Central Institute for Experimental Animals, Kawasaki, Japan; La Jolla Institute of Allergy and Immunology, United States of America

## Abstract

Epstein-Barr virus (EBV) has been implicated in the pathogenesis of rheumatoid arthritis (RA) on the basis of indirect evidence, such as its presence in affected joint tissues, antigenic cross reactions between EBV and human proteins, and elevated humoral and cellular anti-EBV immune responses in patients. Here we report development of erosive arthritis closely resembling RA in humanized mice inoculated with EBV. Human immune system components were reconstituted in mice of the NOD/Shi-*scid*/IL-2Rγ^null^ (NOG) strain by transplantation with CD34^+^ hematopoietic stem cells isolated from cord blood. These humanized mice were then inoculated with EBV and examined pathologically for the signs of arthritis. Erosive arthritis accompanied by synovial membrane proliferation, pannus formation, and bone marrow edema developed in fifteen of twenty-three NOG mice transplanted with human HSC and inoculated with EBV, but not in the nine NOG mice that were transplanted with HSC but not inoculated with EBV. This is the first report of an animal model of EBV-induced arthritis and strongly suggest a causative role of the virus in RA.

## Introduction

A number of observations including those by the authors have suggested the involvement of Epstein-Barr virus (EBV) in the pathogenesis of rheumatoid arthritis (RA)[1], [2], [3], [4], [5], [6], [7]. For example, circulating EBV load is higher in RA patients than in healthy controls [8] and activated CD8-positive cells specific to EBV are commonly seen in RA patients[9]. Further, studies have reported that a large number of T cells specific to EBV-encoded proteins are present in the affected joints of RA patients[10], that interference of suppressor T cells specific to EBV plays a role in RA[11], and that RA patients have abnormally large numbers of EBV-infected B cells in the blood[12]. We have reported on the decreased expression of the gene coding for the signaling lymphocytic activation molecule-associated protein (SAP) (also known as the Src homology 2 domain-containing protein 1A (SH2D1A)) that is supposed to have critical roles in the elimination of EBV-infected B cells by cytotoxic T cells and NK cells [13]. This reduced expression of SAP might lead to the failure of the immune system to eliminate EBV-infected B cells in RA patients [14]. These studies, however, provided only indirect evidence for the involvement of EBV in RA and there have been no published reports on EBV-induced arthritis in experimental animals.

Although model animals for EBV infection are required to examine a causal relationship between EBV and RA, there has been no appropriate animal models suitable for this purpose. EBV can infect only limited primate species and does not infect normal mice. Recently, we developed a humanized mouse model of EBV infection, based on the NOD/Shi-*scid*/IL-2Rγ^null^ (NOG) mouse strain[15], that can reproduce key aspects of human EBV infection, such as lymphoproliferative disorder, asymptomatic persistent infection, and humoral and T cell–mediated immune responses[15]. In this model, where human immune components were reconstituted by transplantation with cord blood-derived CD34^+^ stem cells, inoculation with high-dose EBV (∼1×10^3^ 50% transformation dose [TD_50_]) resulted in the development of lymphoproliferative disorder, whereas inoculation with low-dose virus (<1×10^1^ TD50) tended to cause apparently asymptomatic persistent infection [15]. Immunological analyses of these mice demonstrated the presence of EBV-specific CD8^+^ T cells that inhibit transformation of autologous B lymphocytes by the virus [16]. In the present study, we characterized histopathology of joint tissues obtained from EBV-infected humanized NOG mice and demonstrated erosive arthritis with many features resembling those of RA.

## Materials and Methods

### Ethics Statement and Preparation of humanized mice

NOG mice were obtained from the Central Institute for Experimental Animals (Kawasaki, Japan), and protocols for experiments with NOG mice were approved by the Institutional Animal Care and Use Committee of the National Institute of Infectious Diseases (NIID; Tokyo, Japan) (certification number 206061, 14th. April 2006). Cord blood was obtained from the Tokyo Cord Blood Bank (Tokyo, Japan) after acquiring informed consent from the parents of the donors. Protocols for experiments with human materials were approved by the Institutional Review Boards of the National Research Institute for Child Health and Development (Tokyo, Japan) (certification number 139, 22th. March 2005), the NIID (certification number 1, 17th. October 1997), and the Tokyo Cord Blood Bank (certification number 06-17-02, 18th. August 2006). Isolation of human CD34^+^ HSCs from cord blood using the MACS Direct CD34 Progenitor Cell Isolation Kit (Miltenyi Biotec, Bergisch Gladbach, Germany), their intravenous injection (1×10^4^ to 1.2×10^5^ cells/mouse) into 6- to 10-week-old female NOG mice, and characterization of reconstitution of human hematoimmune system components in these mice were performed as described elsewhere[17]. NOG mice were not irradiated prior to transplantation with CD34^+^ HSCs, because they lived significantly longer after humanization and satisfactory development of human immune system components were observed without irradiation [18]. NOG mice in which human hematoimmune system components were reconstituted are referred to here as humanized NOG (hNOG) mice.

### Analysis on the reconstitution of human lymphoid system components in hNOG mice

Peripheral blood mononuclear cells were isolated weekly from NOG mice following transplantion with human CD34^+^ stem cells and examined for the reactivity with the following antibodies by flow cytometry: FITC-conjugated anti–human CD45 (J.33), CD3 (UCHT1), CD4 (13B8.2), CD19 (J4.119), and CD45RO (UCHL1) (all from Beckman Coulter, Brea, CA); PE-conjugated anti–human CD4 (13B8.2), CD8 (B9.11), CD19 (J4.119), CD45RA (ALB11) (all from Beckman Coulter), and CXCR4 (44717; R&D Systems, Minneapolis, MN); anti–mouse CD45 (YW62.3; Beckman Coulter); ECD-conjugated anti–human CD45 (J.33; Beckman Coulter); and PC5-conjugated anti–human CD8 (T8) and CD14 (Rm052) (all from Beckman Coulter). Flow cytometric analysis was conducted by 2- or 4-color staining using the EpicsXL flow cytometer (Beckman Coulter).

### Experimental EBV infection and quantification of viral DNA

Supernatant fluid of Akata cell culture was prepared as described previously[15] and used as EBV inoculum. EBV dose in 50% transformation dose (TD_50_) was determined by a standard method as described previously[15]. EBV was inoculated intravenously through the tail vein. Peripheral blood EBV DNA load was quantified by real-time polymerase chain reaction (PCR) based on the TaqMan system (Applied Biosystems), as described elsewhere[19]. As a control, nine hNOG mice were left un-infected; among them four mice were inoculated with supernatant fluid of EBV-negative Akata cell culture.

### Histopathology, in situ hybridization (ISH), and immunohistochemistry

hNOG mice were sacrificed 1 to 12 months after inoculation with EBV and their major joints including knees and ankles were removed and fixed in 10% formalin solution. These specimens were embedded in paraffin and stained with hematoxylin-eosin (HE) for histological examinations. For phenotypic analysis of proliferating lymphocytes, immunostaining with the antibodies specific to human CD3 (DAKO, A0452), CD4 (Leica, NCL-CD4-1F6), CD8 (Leica, NCL-CD8-4B11), CD20 (DAKO, M0755) and CD68 (DAKO, M0876) was performed on paraffin sections. EBV was detected by in-situ hybridization (ISH) with EBV-encoded small RNA (EBER) probes (DAKO, Y5200).

### Statistical Analysis

Fisher's exact test was used for categorical data. Analyses were performed using JMP 7.0.2 for Windows (SAS Institute Inc., Cary, NC). All tests were two-tailed, with differences reported as significant when p values were less than 0.01.

## Results

**Figure 1:**
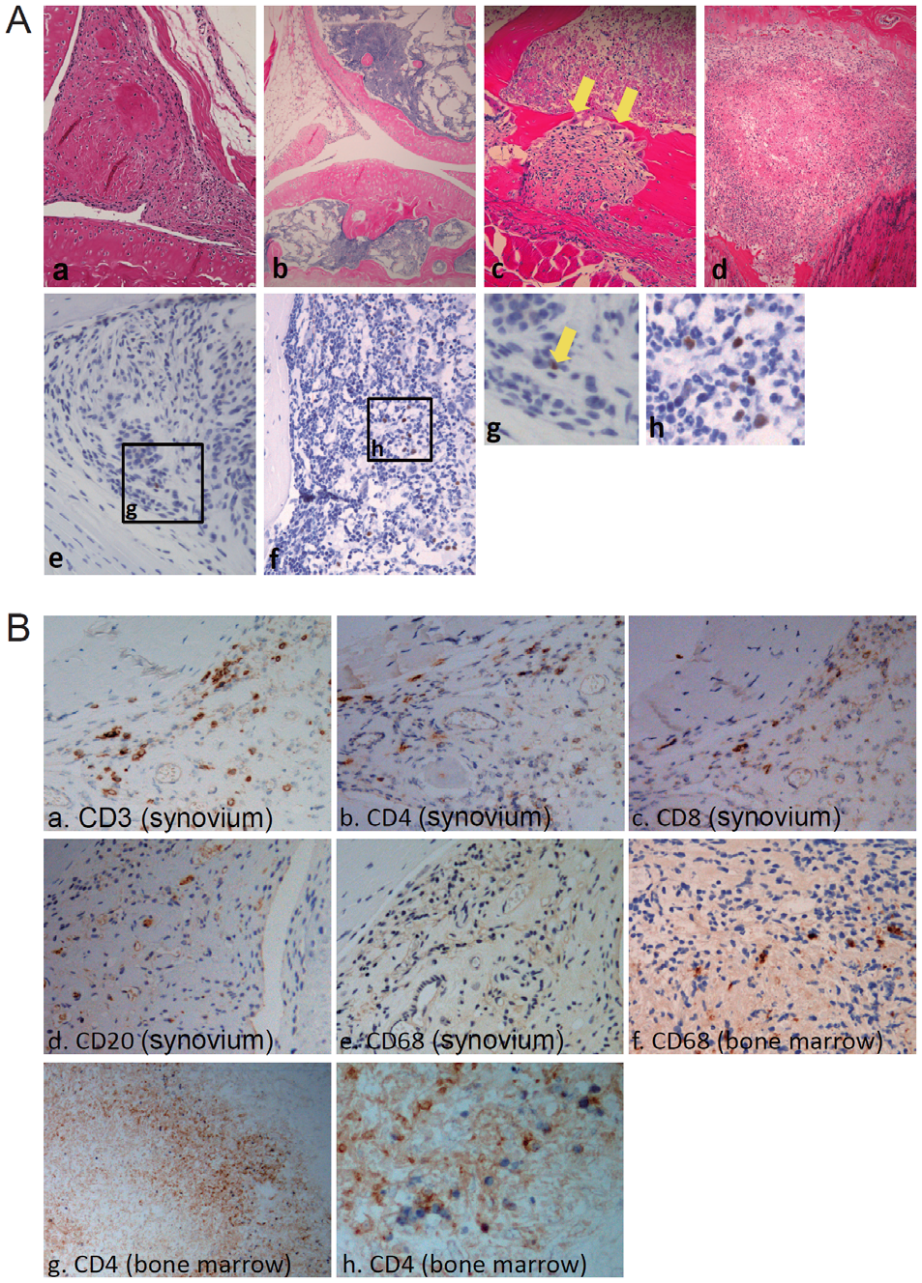
Histopathology of joint and adjacent bone marrow tissues in hNOG mice infected with EBV. A. HE staining and EBER ISH. HE staining of a knee joint in the EBV-infected mouse N70-13 (Table S1), showing synovial proliferation (a): a knee joint in the control mouse N69-1 not infected with EBV (b): a knee joint in the EBV-infected mouse N87-6 (Table S1), showing a pannus-like lesion containing multinuclear giant cells (yellow arrows) (c): and bone marrow near the knee joint of N70-13, showing edema (d). EBER ISH in the synovium of a knee joint in the EBV-infected mouse N79-1 (Table S1), showing few EBV-infected cells (e), and in the bone marrow adjacent to the affected knee joint of the same mouse, demonstrating a number of EBV-infected cells (f). g and h represent further magnification of a portion of e and f, respectively. The yellow arrow indicates an EBER^+^ cell. Original magnification, ×200 (a, c, d, e, f), ×100 (b). B. Immunostaining. Joint (a–e) and adjacent bone marrow tissues (f–h) from the N70-13 mouse were examined for the expression of CD3 (a), CD4 (b, g, h), CD8 (c), CD20 (d), and CD68 (e, f). Original magnification; x200 (a–b), x400 (c–f), x100 (g), x400 (h).

Twenty-three hNOG mice, prepared with CD34^+^ cells isolated from ten different cord blood samples and inoculated with EBV were examined histopathologically for the presence of erosive arthritis. The number of transplanted CD34^+^ cells (0.1–1.2×10^5^ cells), days from transplantation with CD34^+^ cells to inoculation with EBV (106–197 days), dose of EBV inoculated (10^0^–10^3^ TD_50_), days from EBV inoculation to autopsy (26–320 days) for each mouse are described in Table S1. As a control, nine hNOG mice prepared with CD34^+^ cells isolated from three different cord blood samples and not inoculated with EBV were examined similarly (Table S1). Among them, four mice were mock inoculated with culture supernatant of EBV-negative Akata cells. HE staining of major joints including knees and ankles revealed synovial proliferation and infiltration of inflammatory cells in the synovium in 15 of the 23 EBV-infected hNOG mice (65%), whereas none of the nine control hNOG mice showed these signs of arthritis (P = 0.001 by the two-tailed Fisher's exact test) (Fig. 1A and Table 1). Development of arthritis was not dependent on viral dose, because hNOG mice developed arthritis following EBV inoculation at each dose (10^0^, 10^1^, 10^2^, 10^3^ TD_50_) (Table S1). The earliest time point when arthritis was observed was 26 days post-infection and it was seen as late as 320 days post-infection. In a fraction of examined mice, granulation tissue overgrew the bearing surface of the joint and was associated with the breakdown of the articular surface. Furthermore, multinuclear giant cells similar to osteoclasts were seen in the granulation tissue that invaded the bone on the joint edge (Figure 1A). This histology is remarkably similar to the pannus formation seen in erosive arthritis characteristic to RA. In the bone marrow adjacent to inflamed joints, infiltration of activated mononuclear cells generated a histology reminiscent of bone marrow edema characteristic to RA (Fig. 1A).

**Table 1:** Development of arthritis in EBV-infected hNOG mice.

Arthritis	EBV-infected	Un-infected
(+)	15 *	0
(−)	8	9

*p = 0.001, by two-tailed Fisher's exact test.

Immunostaining with monoclonal antibodies revealed a large number of CD3-positive T cells among the proliferating cells in the synovial tissue (Fig. 1B). Both CD4^+^ and CD8^+^ cells were identified. There were also a few CD20^+^ B cells and CD68^+^ macrophages. When the bone marrow adjacent to inflamed joint tissue was examined by immunostaining, CD3-positive cells and CD4-positive cells were detected, while almost no cells exhibited positive CD20 staining (Figure 1B). It should be noted that because NOG mice lack T, B, and NK lymphocytes completely and human-specific antibodies were used here, all lymphocytes detected in hNOG mouse tissues are considered to be of human origin. EBER ISH revealed only few EBV-infected cells in the synovial membrane of affected joints (Fig. 1A), whereas numerous EBV-infected cells were detected in the bone marrow near the affected joints (Fig. 1A).

## Discussion

Erosive arthritis was observed in 15 among the 23 hNOG mice infected with EBV but not in nine control mice that were reconstituted with human immune system components but not inoculated with EBV. The incidence of erosive arthritis is significantly elevated among EBV-infected mice compared with control uninfected mice (P = 0.001 by the two-tailed Fisher's exact test). This erosive arthritis is accompanied by pannus formation, synovial membrane proliferation, inflammatory cell infiltration to the synovium, and bone marrow edema, rendering it closely similar to the tissue of RA. In addition, numerous EBER-positive cells were seen in the edematous bone marrow adjacent to the affected joint. Thus, these results provide the first evidence that EBV can induce erosive arthritis resembling RA in experimental animals. We examined whether anti-cyclic citrullinated peptide (CCP) antibodies and rheumatoid factor (RF), two major markers of RA, were present in the blood of hNOG, but neither was detected.

Few EBER-positive cells were detected in the synovium of affected mouse joints and therefore it is unlikely that EBV-infected cells elicited strong virus-specific immune responses in the synovium and these immune responses triggered aberrant effects damaging the surrounding tissue. However, as numerous CD4^+^ T cells, as well as EBER^+^ cells, were found in the edematous bone marrow adjacent to the affected joint, it is conceivable that migration of inflammatory cells from bone marrow to synovium via ostioles, as Ochi an others suggested, had a role in the initiation of erosive arthritis [20]. It is also possible that inflammatory cytokines produced in bone marrow diffused through the nutrient canal or the nutrient foramen to the synovium and induce the proliferation of synoviocytes and the activation of osteoclasts in the adjacent joint. Significant levels (150–200 pg/ml) of IFN-γ were detected in the plasma of EBV-infected humanized mice. Antigenic cross reaction between EBV proteins and host mouse tissues might have been also involved in the pathogenesis of erosive arthritis in the mice. It should be noted, however, that after rigorous examination we have not detected anti-EBV antibodies in EBV-infected hNOG mice, except for anti-p18*^BFRF3^* (the18-kDa protein encoded by the third rightward open reading frame in the BamHI F fragment) IgM antibody shown in four out of thirty examined mice[15]. We did not detect antibodies to either EBNA1 that cross-reacts with a 62 kDa protein found in the synovium affected by RA [5] or gp110 that cross-reacts with HLA-DR [6], [21]. Antigenic mimicry involving humoral immune responses may thus be unlikely to have a major role in the pathogenesis of erosive arthritis in hNOG mice. In contrast, we observed abundant T-cell response to EBV infection in hNOG mice[15], [16] and it is conceivable that these strong T-cell response has some role in the generation of erosive arthritis.

The present mouse model of erosive arthritis may be an excellent system to investigate the pathogenesis of RA. In this model, it is feasible to remove particular cellular or molecular factors implicated in the pathogenesis of RA by administration of specific antibodies or specific functional antagonists[16]. Analysis on the effects of these antibodies or antagonists will clarify the role of individual cellular and molecular components of the immune system and hence give new insights to the pathogenesis of RA. In a similar approach, this model can also be used to search for molecular and/or cellular targets of novel therapeutics for RA.

## Supporting Information

Table S1 **hNOG mice examined for the development of arthritis.**
(DOC)

## References

[1] Alspaugh MA, Jensen FC, Rabin H, Tan EM (1978). Lymphocytes transformed by Epstein-Barr virus. Induction of nuclear antigen reactive with antibody in rheumatoid arthritis.. J Exp Med.

[2] Billings PB, Hoch SO, White PJ, Carson DA, Vaughan JH (1983). Antibodies to the Epstein-Barr virus nuclear antigen and to rheumatoid arthritis nuclear antigen identify the same polypeptide.. Proc Natl Acad Sci U S A.

[3] Rhodes G, Carson DA, Valbracht J, Houghten R, Vaughan JH (1985). Human immune responses to synthetic peptides from the Epstein-Barr nuclear antigen.. J Immunol.

[4] Rumpold H, Rhodes GH, Bloch PL, Carson DA, Vaughan JH (1987). The glycine-alanine repeating region is the major epitope of the Epstein-Barr nuclear antigen-1 (EBNA-1).. J Immunol.

[5] Fox R, Sportsman R, Rhodes G, Luka J, Pearson G (1986). Rheumatoid arthritis synovial membrane contains a 62,000-molecular-weight protein that shares an antigenic epitope with the Epstein-Barr virus-encoded associated nuclear antigen.. J Clin Invest.

[6] Roudier J, Rhodes G, Petersen J, Vaughan JH, Carson DA (1988). The Epstein-Barr virus glycoprotein gp110, a molecular link between HLA DR4, HLA DR1, and rheumatoid arthritis.. Scand J Immunol.

[7] Takei M, Mitamura K, Fujiwara S, Horie T, Ryu J (1997). Detection of Epstein-Barr virus-encoded small RNA 1 and latent membrane protein 1 in synovial lining cells from rheumatoid arthritis patients.. Int Immunol.

[8] Balandraud N, Meynard JB, Auger I, Sovran H, Mugnier B (2003). Epstein-Barr virus load in the peripheral blood of patients with rheumatoid arthritis: accurate quantification using real-time polymerase chain reaction.. Arthritis Rheum.

[9] Lunemann JD, Frey O, Eidner T, Baier M, Roberts S (2008). Increased frequency of EBV-specific effector memory CD8+ T cells correlates with higher viral load in rheumatoid arthritis.. J Immunol.

[10] Scotet E, David-Ameline J, Peyrat MA, Moreau-Aubry A, Pinczon D (1996). T cell response to Epstein-Barr virus transactivators in chronic rheumatoid arthritis.. J Exp Med.

[11] Tosato G, Steinberg AD, Blaese RM (1981). Defective EBV-specific suppressor T-cell function in rheumatoid arthritis.. N Engl J Med.

[12] Tosato G, Steinberg AD, Yarchoan R, Heilman CA, Pike SE (1984). Abnormally elevated frequency of Epstein-Barr virus-infected B cells in the blood of patients with rheumatoid arthritis.. J Clin Invest.

[13] Filipovich AH, Zhang K, Snow AL, Marsh RA (2010). X-linked lymphoproliferative syndromes: brothers or distant cousins?. Blood.

[14] Takei M, Ishiwata T, Mitamura K, Fujiwara S, Sasaki K (2001). Decreased expression of signaling lymphocytic-activation molecule-associated protein (SAP) transcripts in T cells from patients with rheumatoid arthritis.. Int Immunol.

[15] Yajima M, Imadome K, Nakagawa A, Watanabe S, Terashima K (2008). A new humanized mouse model of Epstein-Barr virus infection that reproduces persistent infection, lymphoproliferative disorder, and cell-mediated and humoral immune responses.. J Infect Dis.

[16] Yajima M, Imadome K, Nakagawa A, Watanabe S, Terashima K (2009). T cell-mediated control of Epstein-Barr virus infection in humanized mice.. J Infect Dis.

[17] Watanabe S, Terashima K, Ohta S, Horibata S, Yajima M (2007). Hematopoietic stem cell-engrafted NOD/SCID/IL2Rgamma null mice develop human lymphoid systems and induce long-lasting HIV-1 infection with specific humoral immune responses.. Blood.

[18] Watanabe S, Ohta S, Yajima M, Terashima K, Ito M (2007). Humanized NOD/SCID/IL2Rgamma(null) mice transplanted with hematopoietic stem cells under nonmyeloablative conditions show prolonged life spans and allow detailed analysis of human immunodeficiency virus type 1 pathogenesis.. J Virol.

[19] Kimura H, Morita M, Yabuta Y, Kuzushima K, Kato K (1999). Quantitative analysis of Epstein-Barr virus load by using a real-time PCR assay.. J Clin Microbiol.

[20] Ochi T, Hakomori S, Adachi M, Owaki H, Okamura M (1988). The presence of a myeloid cell population showing strong reactivity with monoclonal antibody directed to difucosyl type 2 chain in epiphyseal bone marrow adjacent to joints affected with rheumatoid arthritis (RA) and its absence in the corresponding normal and non-RA bone marrow.. J Rheumatol.

[21] Roudier J, Petersen J, Rhodes GH, Luka J, Carson DA (1989). Susceptibility to rheumatoid arthritis maps to a T-cell epitope shared by the HLA-Dw4 DR beta-1 chain and the Epstein-Barr virus glycoprotein gp110.. Proc Natl Acad Sci U S A.

