# Supplementary material for: Epstein-Barr Virus Induces Erosive Arthritis in Humanized Mice
Source: PLoS One. 2011 Oct 19;6(10):e26630. doi: 10.1371/journal.pone.0026630 (PMC3197576; doi:10.1371/journal.pone.0026630)
Supplement: Table S1 — hNOG mice examined for the development of arthritis. (DOC) [file pone.0026630.s001.doc]

Table S1. hNOG mice examined for the development of arthritis

| Mouse | Transplanted CD34+ cells  (×10^5^ cells) | Days from transplantation to infection | EBV dose | Days from infection to autopsy | Arthritis |
| --- | --- | --- | --- | --- | --- |
| N70-13 | 0.61 | 197 | 1.0×10^3^ | 61 | (+) |
| N75-1 | 0.89 | 106 | 4.0×10^0^ | 161 | (+) |
| N75-3 | 0.89 | 106 | 1.0×10^1^ | 320 | (+) |
| N78-6 | 0.875 | 145 | 8.0×10^2^ | 164 | (－) |
| N78-8 | 0.875 | 145 | 8.0×10^2^ | 164 | (－) |
| N79-1 | 0.86 | 112 | 4.0×10^0^ | 97 | (+) |
| N81-2 | 0.435 | 128 | 4.0×10^0^ | 291 | (－) |
| N81-3 | 0.435 | 128 | 4.0×10^0^ | 291 | (－) |
| N82-2 | 0.4 | 155 | 4.0×10^0^ | 75 | (－) |
| N82-4 | 0.4 | 155 | 4.0×10^0^ | 41 | (+) |
| N83-1 | 1.12 | 174 | 4.0×10^0^ | 30 | (+) |
| N83-3 | 0.435 | 174 | 4.0×10^0^ | 38 | (+) |
| N85-4 | 0.58 | 139 | 4.0×10^0^ | 99 | (+) |
| N87-3 | 0.5 | 124 | 1.0×10^1^ | 131 | (－) |
| N87-4 | 0.5 | 124 | 1.0×10^1^ | 71 | (+) |
| N87-5 | 0.5 | 124 | 4.0×10^0^ | 97 | (－) |
| N87-6 | 0.5 | 124 | 4.0×10^0^ | 90 | (+) |
| N87-7 | 0.5 | 124 | 4.0×10^0^ | 97 | (+) |
| N90-10 | 0.73 | 177 | 1.0×10^2^ | 26 | (+) |
| N90-13 | 0.73 | 177 | 1.0×10^2^ | 26 | (+) |
| N90-15 | 0.73 | 177 | 1.0×10^2^ | 26 | (+) |
| N90-17 | 0.73 | 177 | 1.0×10^2^ | 26 | (－) |
| N90-19 | 0.73 | 177 | 1.0×10^2^ | 26 | (+) |
| N69-1 | 0.42 | NA^1^ | NA^1^ | - | (－) |
| N69-3 | 0.42 | NA^1^ | NA^1^ | - | (－) |
| N69-4 | 0.42 | NA^1^ | NA^1^ | - | (－) |
| N87-1 | 0.5 | NA^1^ | NA^1^ | - | (－) |
| N87-8 | 0.5 | NA^1^ | NA^1^ | - | (－) |
| NN8-10^2^ | 0.5 | NA^1^ | NA^1^ | - | (－) |
| NN8-11^2^ | 0.5 | NA^1^ | NA^1^ | - | (－) |
| NN8-12^2^ | 0.5 | NA^1^ | NA^1^ | - | (－) |
| NN8-13^2^ | 0.5 | NA^1^ | NA^1^ | - | (－) |

^1^Not applicable.

^2^Culture supernatant of EBV-negative Akata cells was used as inoculum.
